# Seropositivity of antibody to hepatitis B core antigen among hepatitis B surface antigen-negative vaccinated individuals aged 5–12 years in North West Ethiopia

**DOI:** 10.1016/j.heliyon.2024.e40107

**Published:** 2024-11-02

**Authors:** Adane Adugna, Deresse Sinamaw, Temesgen Baylie, Mamaru Getinet, Aysheshim Belaineh Haimanot, Gashaw Azanaw Amare, Habtamu Belew, Zigale Hibstu, Desalegn Abebaw, Abebe Fenta, Muluken Getinet, Dagmawi Abiy, Agenagnew Ashagre, Mohammed Jemal

**Affiliations:** a Department of Medical Laboratory Science, College of Health Sciences, Debre Markos University, Debre Markos, Ethiopia; b Department of Biomedical Sciences, School of Medicine, College of Health Sciences, Debre Markos University, Debre Markos, Ethiopia; c Department of Public Health, College of Health Sciences, Debre Markos University, Debre Markos, Ethiopia; d Department of Pathology, School of Medicine, College of Health Sciences, Debre Markos University, Debre Markos, Ethiopia; e Department of Internal Medicine, School of Medicine, College of Health Sciences, Debre Markos University, Debre Markos, Ethiopia; f Department of Medical Laboratory Science, College of Health Sciences, Woldiya University, Woldiya, Ethiopia

**Keywords:** Seropositivity, HBsAg, Anti-HBc, Vaccinated individuals, North West Ethiopia

## Abstract

**Background:**

Infection with the hepatitis B virus (HBV) is still a major global public health concern, especially in Ethiopia. Evidence suggests that some children in Ethiopia who have received hepatitis B vaccinations are still contracting HBV.

**Objective:**

The main aim of this study was to detect antibodies to hepatitis B core antigen (anti-HBc) among vaccinated hepatitis B surface antigen (HBsAg)-negative individuals in North-West Ethiopia.

**Methods and materials:**

A community-based cross-sectional study was conducted among 158 children aged 5–12 years from April 2021 to November 2021. A simple random sampling technique was used to recruit study participants. After 3–5 ml of venous blood was drawn from each study participant, a serum sample was utilized to determine hepatitis B surface antigen (HBsAg) and anti-hepatitis B core antibodies (anti-HBc) by enzyme-linked immunosorbent assay (ELISA). Logistic regression with a 95 % CI was used to show the statistical association.

**Results:**

The total seropositivity of anti-HBc among vaccinated HBsAg-negative participants was 8/158 (5.1 %) (95 % CI: 2.0–9.0). Multivariable logistic regression revealed that children who had a previous history of blood transfusion were six times (AOR: 6.135, 95 % CI: 4.063, 10.752) (P < 0.006) more likely to develop anti-HBc. Moreover, children who had a previous history of surgery were five times (AOR: 5.116, 95 % CI: 3.123, 8.718) (P < 0.002) more likely to become anti-HBc seropositive.

**Conclusion:**

There was a significant seroprevalence of anti-HBc in our study area, suggesting possible exposure to the virus despite immunization.

## 1 Introduction

Hepatitis B virus (HBV) infection is a serious worldwide health concern. It is projected that 254 million individuals globally have a persistent infection with HBV. Moreover, there are 1.2 million new cases of hepatitis B infections, and about 6000 new cases of the disease occur every day [1]. Frequent surveillance of seroprevalence data contributes to public health policies intended to lower the worldwide incidence of hepatitis B virus infection [2].

There have been initiatives to stop HBV infection via regular immunization campaigns. All infants should receive a hepatitis B vaccination at birth and finish the course of shots by the time they are one year old, according to recommendations made by the World Health Organization (WHO) [3]. The National Expanded Program for Immunization (EPI) in Ethiopia included HBV immunization as a pentavalent in 2007. The HBV vaccine is administered at 6, 10, and 14 weeks following delivery, per EPI regimens [4].

Even with these initiatives, attaining universal coverage and getting rid of new infections still present difficulties. Ethiopia is home to high endemicity of hepatitis B because of several variables, including low awareness, inadequate healthcare facilities, restricted access to immunization programs, and cultural traditions that may facilitate the spread of the disease.

It has been demonstrated that the hepatitis B vaccination is quite successful at preventing HBV infection [5].

A major factor in the decline in new pediatric infections has been the inclusion of the universal hepatitis B vaccination in the national vaccination schedule [6]. To further lower the prevalence of HBV infection in Ethiopia, nevertheless, considerable work remains to be done despite these initiatives [7]. The HBV immunization campaign in Ethiopia has proven successful in decreasing the rate of transmission among children who have received vaccinations. However, to provide long-term protection against HBV infection, continuing observation and assessment are required, as vaccinated children may still get breakthrough infections [8].

A variety of reasons, including insufficient vaccination response, exposure to elevated viral levels, or novel HBV strains not protected by the vaccine, could be the cause of these breakthrough infections [9].

An essential component in tracking the efficacy of the hepatitis B vaccine and evaluating the immune system's reaction to the virus is the identification of hepatitis core antigen antibodies (anti-HBc) in children who have received vaccinations [10].

Whether an HBV infection is resolved or still active, anti-HBc develops and usually lasts a lifetime. Anti-HBc does not develop in those whose protection against HBV comes from a vaccination. The presence of anti-HBc shows previous or ongoing HBV infection within an undefined time frame [11]. Medical professionals and public health authorities keep an eye on and look into situations in which immunized children test positive for anti-HBc antibodies. This makes it easier to spot any possible vaccination efficacy gaps or places that can benefit from further efforts [12].

Frequent anti-HBc antibody testing among children who have received vaccinations can help inform public health initiatives targeted at lowering the spread of hepatitis B and its related consequences among youth. It can also yield important data on vaccination efficacy rates [8].

Moreover, there is no published data on the seroprevalence of anti-HBc among vaccinated children in the study area who test negative for the hepatitis B surface antigen (HBsAg). Hence, this study aimed to detect the antibodies to hepatitis B core antigen (anti-HBc) among vaccinated HBsAg-negative individuals in North-West Ethiopia.

## 2 Materials and methods

**Study design, period, and setting:** A community-based cross-sectional study was carried out among vaccinated hepatitis B surface antigen-negative individuals in Debre Markos Town, North-West Ethiopia, from April to November 2021.

**Inclusion and exclusion criteria:** The source population for our study included all children between ages five to twelve years who had received all recommended doses of the hepatitis B vaccine in the study area, and those who were negative for the hepatitis B surface antigen and satisfied the eligibility criteria were taken as the study population.

The study included all children who received the complete dose of the hepatitis B vaccination (three doses), who gave their consent to participate in the study, and whose parents or legal guardians volunteered to provide written informed assent. The study did not include participants who did not receive the recommended doses of the hepatitis B vaccine or whose parents were too unwell to provide assent for their children and children who declined to provide blood samples.

### 2.1 Sample size determination and sampling techniques

The sample size was determined using 89 % of the effectiveness of the HBV vaccine observed in a study conducted in Ethiopia [13]. In this calculation, we considered p as the proportion (0.89), q as 1-p, and d as the margin of error (0.05). It was performed with a 95 % confidence interval, corresponding to a Z-value of 1.96, and included a 10 % non-response rate.

$$n=\frac{{\left(\frac{Za}{2}\right)}^{2}pq}{d^{2}}\;n={\left(1.96\right)}^{2}/\;{\left(0.05\right)}^{2}\;*0.89*0.11=150+10\%*150$$

The sample size (n) = 165.

Using a simple random sampling method, 165 study participants were selected. However, after laboratory analysis of HBsAg, only 158/165 study participants who were negative for HBsAg were used for statistical analysis; the remaining 7 study participants were excluded from the statistical analysis as they were positive for HBsAg [14].

### 2.2 Data collection and laboratory procedures

Independent factors, including socioeconomic characteristics and clinical variables, were gathered using a pretested structured questionnaire following the acquisition of legal guardians' informed written assent, and then 3–5 ml of blood samples were taken from all study participants. Following centrifugation, the serum was kept at −20 °C until laboratory examination. Using a qualitative sandwich enzyme-linked immunosorbent assay [BIO-RAD, Monolisa, France], HBsAg and anti-HBc were detected. Anti-HBc was detected using the Monolisa™ anti-HBc PLUS ELISA kit. The microplate wells were pre-coated with hepatitis B core antigen (HBcAg). Subsequently, 100 μl of serum samples were added to each well. Antibodies labeled with peroxidase were then introduced to bind with human IgG and IgM, forming an antigen-antibody-enzyme-labeled antibody complex. After stopping the reaction, the ELISA results were read at 450, 620, and 700 nm. Each testing procedure adhered to the manufacturer's instructions. To mitigate false-positive results, standards for the anti-HBc ELISA kit were checked against known positive and negative control samples according to the kit protocol before conducting the actual laboratory analysis. The laboratory results were read, reported, and interpreted based on the kit protocol and appropriate reference ranges.

**Data processing and analysis:** Data were entered into Epidata software version 3.1 and exported to SPSS software version 25 for analysis once the completeness of the data was verified. Tables were used to display the results. To demonstrate the relationship between the determinant and outcome variables, binary logistic regression was utilized. A p-value of less than 0.25 in bivariate logistic regression indicated prospects for multivariate logistic regression. Adjusted odds ratios (AOR) with 95 % confidence intervals were calculated using multivariate logistic regression, and a p-value of less than 0.05 was deemed statistically significant.

## 3 Ethical consideration and consent to participate

The research and ethics committee of Debre Markos University, College of Health Science, approved this work ethically and provided reference number HSC/R/C/Ser/PG/CO/277/12/2021.

Oral consent and written informed assent were obtained from each study participant and their legal guardian, respectively.

## 4 Results

### 4.1 Socio-demographic characteristics

A total of 158 individuals in the age range of 5–12 years were enrolled in the current study. The median ages of the study participants were 8.00 ± 2.6. Moreover, the majority of the study participants (53.9 %) were males (Table 1).

**Table 1:** Socio-demographic characteristics of study participants and their legal guardians.

Socio-demographic variables	Age category	Frequency	Percent
Age of child	5–7	70	44.3
	8–10	47	29.7
	11–12	41	25.9
Child's sex	Male	83	52.5
	Female	75	47.5
Educational status (for parents)	Iliterate	23	14.6
	Litrate	135	85.4
Occupation (for parents)	Farmer	20	12.7
	Government employer	42	26.6
	Private	23	14.6
	Daily laborer	15	9.5
	Housewife	28	17.7
	Merchant	30	19.0
Total		158	100.0

Seropositivity of antibodies to hepatitis B core antigen (anti-HBc) among study participants with their respective socio-demographic characteristics.

The total seropositivity of anti-HBc among all study participants was 8/158 (5.1 %) (95 % CI: 2.0–9.0). There were equal distributions (50.0 %) of anti-HBc positivity in both sexes. Furthermore, of the total anti-HBc study participants, half were in the age group of ≥11 years (Table 2).

**Table 2:** The seropositivity of anti-HBc with participants’ socio-demographic variables.

Variables	Category	Antibody to core antigen		
		Positive	Negative	Total
Age of the child	5–7	2(25.0 %)	68(45.3 %)	70
	8–10	2(25.0 %)	45(30.0 %)	47
	11–12	4(50.0 %)	37(24.7 %)	41
Child's sex	Male	4(50.0 %)	79(52.7 %)	83
	Female	4(50.0 %)	71(47.3 %)	75
Educational status	Iliterate	1(12.5 %)	22(14.7 %)	23
	Literate	7(87.5 %)	128(85.3 %)	135
Occupation	Farmer	2(25.0 %)	18(12.0 %)	20
	Government employer	0(0.0 %)	42(28.0 %)	42
	Private	2(25.0 %)	1(14.0 %)	23
	Daily laborer	1(12.5 %)	14(9.3 %)	15
	Housewife	1(12.5 %)	27(18.0 %)	28
	Merchant	2(25.0 %)	28(18.7 %)	30

### 4.2 Seropositivity of anti-HBc and its distribution with participants’ clinical variables

There was equal distribution (50.0 %) of anti-HBc among children with a previous history of hospital admission, circumcision, and surgery (Table 3).

**Table 3:** Anti-HBc seropositivity by clinical variables.

Clinical variables	Category	Antibody to core antigen		
		Positive	Negative	Total
HBV status of the child's mother	Yes	1(12.5 %)	1(0.7 %)	2
	No	7(87.5 %)	149(99.3 %)	156
Child's BMI	Normal	8(100.0 %)	118(78.7 %)	126
	Uw	0(0.0 %)	17(11.3 %)	17
	Ow	0(0.0 %)	15(10.0 %)	15
History of hospital admissions	Yes	4(50.0 %)	15(10.0 %)	19
	No	4(50.0)	135(90.0 %)	139
History of blood transfusions	Yes	3(37.5 %)	1(0.7 %)	4
	No	5(62.5 %)	149(99.3 %)	154
Previous exposure to injectable medications	Yes	1(12.5 %)	11(7.3 %)	12
	No	7(87.5 %)	139(92.7 %)	146
Previous exposure to circumcision	Yes	4(50.0 %)	65(43.3 %)	69
	No	4(50.0 %)	85(56.7 %)	89
Previous exposure to body piercing	Yes	1(12.5 %)	19(12.7 %)	20
	No	7(87.5 %)	131(87.3 %)	138
Previous exposure to tattooing	Yes	2(25.0 %)	12(8.0 %)	14
	No	6(75.0 %)	138(92.0 %)	144
Previous history of dialysis	Yes	1(12.5 %)	1(0.7 %)	2
	No	7(87.5 %)	149(99.3 %)	156
Previous history of surgery	Yes	4(50.0 %)	1(0.7 %)	5
	No	4(50.0 %)	149(99.3 %)	153
Previous history of dental extraction	Yes	3(37.5 %)	7(4.7 %)	10
	No	5(62.5 %)	143(95.3 %)	148

NB: % = percentile, BMI=Body Mass Index, Normal weight BMI = 5th-85th%ile, Underweight (Uw) = <5th%ile, Overweight (Ow) = 86 th-95th%ile.

### 4.3 Factors associated with anti-HBc seropositivity

In multiple logistic regression, children who had a previous history of blood transfusion were six times (AOR: 6.135, 95 % CI: 4.063, 10.752) (P < 0.006) more likely to develop anti-HBc compared to those without a history of blood transfusion. Moreover, children with a previous history of surgery were five times (AOR: 5.116, 95 % CI: 3.123, 8.718) (P < 0.002) more likely to become anti-HBc seropositive compared to their counterparts (Table 4).

**Table 4:** Bivariate and multivariate logistic regression of determinants for anti-HBc seropositivity.

Variables	Category	Total anti-body to core antigen					
		Positive	Negative	COR(95%CI)	P-V	AOR(95%CI)	P-V
Mother's history of HBV	Yes	1(12.5 %)	1(0.7 %)	8.29(7.203,10.042)	0.037∗	4.773(3.001,11.128)	0.889
	No	7(87.5 %)	149(99.3 %)	Ref		Ref	
Previous history of hospital admission	Yes	4(50.0 %)	15(10.0 %)	9.00(8.038–12.235)	0.004∗	3.988 (2.063, 7.254)	0.697
	No	4(50.0 %)	135(90.0 %)	Ref		Ref	
Previous history of blood transfusion	Yes	3(37.5 %)	1(0.7 %)	8.000(7.853,10.409)	0.000∗	6.135(4.063, 10.752)	0.006∗∗
	No	5(62.5 %)	149(99.3)	Ref		Ref	
Previous exposure to body tattooing	Yes	2(25.0 %)	12(8.0 %)	3.833(2.696,6.100)	0.123∗	7.114(5.001, 15.341)	0.389
	No	6(75.0 %)	138(92.0 %)	Ref		Ref	
Previous history of dialysis	Yes	1(12.5 %)	1(0.7 %)	11.286(4.203,12.764)	0.037∗	6.368(4.162,11.204)	0.545
	No	7(87.5 %)	149(99.3 %)	Ref		Ref	
Previous history of Surgery	Yes	4(50.0 %)	1(0.7 %)	10.000(8.439,16.040)	0.000∗	5.116(3.123, 8.718)	0.002∗∗
	No	4(50.0 %)	149(99.3 %)	Ref		Ref	
Previous history of dental extraction	Yes	3(37.5 %)	7(4.7 %)	3.257(2.426–9.237)	0.002∗	6.595(5.275, 12.398)	0.244
	No	5(62.5 %)	143(95.3 %)	Ref		Ref	

NB: ∗: Candidate variable for multivariate analysis at P < 0.25 and ∗∗: significant variable by the multivariate analysis at P < 0.05, COR: crude odds ratio, AOR: adjusted odds ratio, CI: confidence interval, P-V: p-value, Ref: reference.

## 5 Discussion

The issue at hand is the significant worldwide prevalence of childhood hepatitis B virus infection. This virus still affects a large number of children despite efforts to stop it from spreading through vaccination programs, screening procedures, and public health initiatives [15].

On the other hand, it is a worrying issue that children in Ethiopia who have received vaccinations are infected with the hepatitis B virus. Even in children who have received full doses of the hepatitis B vaccine, infections still occur [6].

Children who received full doses of the hepatitis B vaccine may test positive for anti-HBc antibodies for various reasons. One reason is prior exposure to hepatitis B virus (HBV) before vaccination. In such cases, although the vaccination provides protection against HBV, these children may still have detectable anti-HBc antibodies from a previous infection [16]. Another reason for hepatitis B vaccine failure is the inability to establish adequate protection against HBV, even after receiving all recommended doses. This may occur due to factors such as the individual's age at the time of vaccination or specific medical conditions that hinder their ability to mount a sufficiently strong immune response [17].

In other words, vaccinated children are occasionally still susceptible to contracting hepatitis B after receiving their vaccination. This is referred to as a breakthrough infection and may cause HBc-specific antibodies to emerge [18]. When anti-HBc is present in vaccinated individuals who test negative for HBsAg, it may be a sign of natural immunity resulting from prior viral exposure [19]. On the other hand, knowing how common anti-HBc is in this population may help shed light on potential protection gaps and the efficacy of hepatitis B vaccination campaigns.

In other words, assessing a person's immunological status and risk of developing an ongoing HBV infection can be aided by this information. Furthermore, the HBsAg marker should be taken into account when interpreting the relevance of anti-HBc findings. Depending on the population and region, there can be differences in the overall prevalence of anti-HBc in vaccinated HBsAg-negative children [20]. In the current study, we focused exclusively on children aged 5–12 years, as the hepatitis B vaccination program for children was initiated in the country in 2007 and specifically in our study area in 2009. Consequently, we did not include children greater than twelve years of age. Additionally, we included children aged greater than or equal to five years to evaluate how HBV vaccination affects anti-HBc seropositivity as the children's age increases.

In the present study, the overall seropositivity of total antibody to hepatitis B core antigen (anti-HBc) among vaccinated hepatitis B surface antigen (HBsAg) negative children was 8/158 (5.1 %) (95 % CI: 2.0–9.0). This high seropositivity rate may be explained by several factors, such as ineffective vaccinations resulting from poor handling or storage, exclusion of birth doses of vaccinations, diminishing immunity over time, or possible transmission through intimate contact with sick people [21].

This result is similar to earlier findings from Gondar (6.3 %) [22] and Addis Abeba (5.6 %) [23]. This finding is also consistent with findings from other nations, such as Brazil (4.4 %) [24], Thailand (5.5 %) [25], Italy (3.3 %) [26], Iran (2.1 %) [27], and Egypt (2.0 %) [28]. However, the present anti-HBc seropositivity is comparatively higher than that of earlier research from Jimma (1.1 %) [29], Senegal (1.5 %) [30], and Egypt (0.11 %) [31]. Conversely, greater results were noted from Hawassa city (19.5 %) [32] and another nation; Gambia (10.2 %) [33].

These variations might be due to different factors, like the efficacy of vaccination. The main consideration is the hepatitis B vaccine's efficacy. The vaccination's effectiveness and longevity of protection can differ based on several variables, including the individual's variability in immunological response and the timing of the injection [34]. Due to specific medical illnesses or immunodeficiency diseases, some people may not have a strong enough immune response following vaccination, which could result in lower levels of protective antibodies or a shorter duration of protection against HBV infection. In addition, the degree and durability of immunity conferred by a vaccine may be impacted by administration delays during the advised pediatric immunization schedule [35].

Moreover, viral variations and resistant modifications may affect the effectiveness of the hepatitis B vaccine despite complete vaccination. In other words, in rare instances where circulating strains of HBV contain mutations that confer resistance or viral strains that are not effectively neutralized by the antibodies in current vaccines, this might potentially result in breakthrough infection [36].

In the present study, half of the anti-HBc-positive children (50.0 %) were ≥11 years. This is due to increased horizontal exposure episodes as children's age probably causes the higher anti-HBc prevalence in older children. An additional factor to take into account would be older children's declining anti-HBs (waning immunity), which would raise their risk of HBV infection [37]. Their immune systems have had more chances to interact with and fight off the virus as they get older, which increases the chance that they will develop anti-HBc antibodies [38]. Furthermore, even if older children were effectively vaccinated against hepatitis B, they may have been exposed to the virus before vaccination, which could result in the formation of anti-HBc antibodies [39].

In this study, associated factors were also assessed, and there were some independent variables associated with anti-HBc seropositivity in vaccinated HBsAg-negative children. Among these predictors, previous history of blood transfusion and surgery were identified as important determinants for anti-HBc seropositivity. Children who had a previous history of blood transfusion were six times more likely to be anti-HBc seropositive than those who had no history of blood transfusion. In addition, children with a previous history of surgery had a five times higher risk of being anti-HBc seropositive as compared to their counterparts.

### 5.1 Limitations of the study

In the current study, only HBsAg and anti-HBc antibodies were detected. Other HBV infection markers like hepatitis B envelope antigen (HBeAg) and anti-HBeAg were not tested due to limited resources. Therefore, the rate of viral replication and degree of infectivity were not determined. Detection and quantification of HBV DNA for positive samples were not done since HBV primers and kits were not available. Levels of anti-HBs antibodies were not also determined due to limited resources. We did not evaluate the child's exposure to HBV infection before vaccination due to a lack of information and resource constraints. Moreover, we have only assessed the HBV status of the child's mother. The HBV status of the child's father and other family members has not been evaluated due to resource and time constraints. In addition, this study was conducted with a small sample size, which may affect the statistical power and the generalizability of the findings.

## 6 Conclusions and recommendations

Among vaccinated children with surface antigen (HBsAg) negativity, the study discovered a significant prevalence of hepatitis B core antibody (anti-HBc) positivity in the study area, suggesting possible exposure to the virus despite immunization. This suggests that even though these children had vaccinations, they had still been exposed to the hepatitis B virus.

Moreover, a history of blood transfusion and surgery were identified as significant determinants of anti-HBc seropositivity. The current findings are concerning because they point to a possible weakness in the present immunization program's ability to shield children from contracting hepatitis B. It draws attention to the necessity of more research into the variables behind this high seropositivity rate among those who have received vaccinations.

Ethiopia's public health officials should assess and resolve these problems by carrying out surveillance studies on the effectiveness of vaccines and, if required, putting preventative measures in place like booster shots. In addition, healthcare workers should properly screen blood products before transfusions. Furthermore, they should also properly sterilize and disinfect medical devices during patient care to prevent horizontal transmissions.

## CRediT authorship contribution statement

**Adane Adugna:** Writing – review & editing, Writing – original draft, Validation, Software, Methodology, Investigation, Formal analysis, Data curation, Conceptualization. **Deresse Sinamaw:** Writing – review & editing, Writing – original draft, Supervision, Conceptualization. **Temesgen Baylie:** Writing – review & editing, Writing – original draft, Visualization. **Mamaru Getinet:** Writing – review & editing, Writing – original draft, Visualization, Validation, Supervision. **Aysheshim Belaineh Haimanot:** Writing – review & editing, Writing – original draft. **Gashaw Azanaw Amare:** Writing – review & editing, Writing – original draft, Software. **Habtamu Belew:** Writing – review & editing, Writing – original draft, Visualization, Data curation. **Zigale Hibstu:** Writing – review & editing, Writing – original draft, Visualization. **Desalegn Abebaw:** Writing – review & editing, Writing – original draft, Validation, Supervision, Investigation. **Abebe Fenta:** Writing – review & editing, Writing – original draft, Visualization, Validation. **Muluken Getinet:** Writing – review & editing, Writing – original draft, Visualization, Validation. **Dagmawi Abiy:** Writing – review & editing, Writing – original draft, Visualization, Validation. **Agenagnew Ashagre:** Writing – review & editing, Writing – original draft, Visualization, Validation, Conceptualization. **Mohammed Jemal:** Writing – review & editing, Writing – original draft, Visualization, Validation, Supervision, Software, Methodology, Investigation, Conceptualization.

## Informed consent statement

Each participant provided their consent, while written informed assent was given by the parents or legal guardians of the participants.

## Ethical approval statement

The Debre Markos University, College of Health Science Institutional Review Board gave its approval to the study, which was carried out in conformity with the Declaration of Helsinki (Protocol number Ref: HSC/R/C/Ser/PG/CO/277/12/2021).

## Consent to publication

Not applicable.

## Funding

Not applicable.

## Declaration of competing interest

The authors declare that they have no known competing financial interests or personal relationships that could have appeared to influence the work reported in this paper.

## Data Availability

Data will be made available on request.
